# Optimal Timing for Primary Early Endoscopic Dacryocystorhinostomy in Acute Dacryocystitis

**DOI:** 10.3390/jcm10102161

**Published:** 2021-05-17

**Authors:** Jae Yun Sung, Ju Mi Kim, Jae Yul Hwang, Kyoung Nam Kim, Jaeyoung Kim, Sung Bok Lee

**Affiliations:** 1 Department of Ophthalmology, Chungnam National University Sejong Hospital, Sejong 30099, Korea; ssungjy@naver.com; 2 Department of Ophthalmology, Chungnam National University College of Medicine, Daejeon 35015, Korea; jjukkum2@naver.com (J.M.K.); emperor1119@hanmail.net (J.Y.H.); kkn9901700@hanmail.net (K.N.K.); scullism@gmail.com (J.K.)

**Keywords:** acute dacryocystitis, endoscopic dacryocystorhinostomy, surgery, timing

## Abstract

Purpose: To evaluate the surgical outcomes of primary early endoscopic dacryocystorhinostomy (EnDCR) in acute dacryocystitis (AD) and to determine the optimal timing for surgery. Methods: A retrospective review of medical records was performed on consecutive patients who underwent primary early EnDCR (within 1 week) for AD between May 2010 and June 2020 (AD group) and an age- and gender-matched control group of NLDO patients who underwent EnDCR (non-AD group). The primary outcome measures were the surgical outcomes at the final follow-up examination. The secondary outcome measure was the clinical course of AD patients. Subgroup analysis was performed to determine the optimal timing of surgery by comparing the outcomes of very early EnDCR (within 3 days) and those of early EnDCR (between 4 and 7 days). Results: Forty-one patients were included in the AD group and 82 patients in the non-AD group. The anatomical and functional success rates were 87.8% and 82.9% in the AD group, and 91.5% and 84.1% in the non-AD group, which were not significantly different between the two groups (*p* = 0.532 and *p* = 0.863). In the AD group, the mean times for pain relief and resolution of swelling after surgery were 2.4 and 6.5 days after surgery, respectively. In the subgroup analysis according to the timing of surgery, the time for symptom resolution after diagnosis, the length of hospital stays, and the duration of antibiotic treatments were significantly shorter after very early EnDCR (all ps < 0.05), whereas the surgical outcomes were not different between the two groups (*p* = 1.000). Conclusions: Primary early EnDCR is a safe and effective procedure for the treatment of AD. In particular, very early EnDCR performed within 3 days leads to faster recovery and shortens the course of antibiotic treatment.

## 1. Introduction

Acute dacryocystitis (AD) is an inflammation of the lacrimal sac that usually occurs secondary to nasolacrimal duct obstruction (NLDO) and is characterized by acute pain, swelling, and erythema in the medial canthal area [1,2]. The conventional treatments include warm compresses, systemic antibiotics, and percutaneous abscess drainage followed by external dacryocystorhinostomy (ExDCR) after resolution of inflammation [2,3,4]. However, the limitations of the conventional treatments include prolonged and recurrent infections, adverse effects of the long-term use of systemic antibiotics, and cutaneous fistula formation [5]. In addition, delayed ExDCR results in cutaneous scarring, disruption of the lacrimal pump, and a risk of surgical failure because of synechiae and granulation tissue within the lacrimal sac [2,6,7]. Recently, endoscopic dacryocystorhinostomy (EnDCR) has emerged as a primary treatment option for AD, with advantages including rapid resolution of inflammatory symptoms and potential economic benefits from shorter hospital stays, shorter antibiotics course, and fewer procedures [3,6,8,9,10]. Recent studies have reported reasonable outcomes of primary EnDCR in patients with AD. The success rate of EnDCR has varied from 81.8% to 94.4% [2,3,9,11]. However, in these previous studies, the time from diagnosis to operation varied from 3 days to 2 weeks and the sample sizes were small which limited the effectiveness of early surgical intervention in AD.

The purpose of this study was to evaluate the surgical outcomes of early EnDCR (within 1 week after diagnosis) in the primary treatment of AD and to report the clinical course of the patients. In addition, to assess the effect of the timing of surgery, a subgroup analysis was performed. The outcomes of very early EnDCR (within 3 days after diagnosis) and early EnDCR (4 to 7 days after diagnosis) were compared.

## 2. Methods

**Figure 1:**
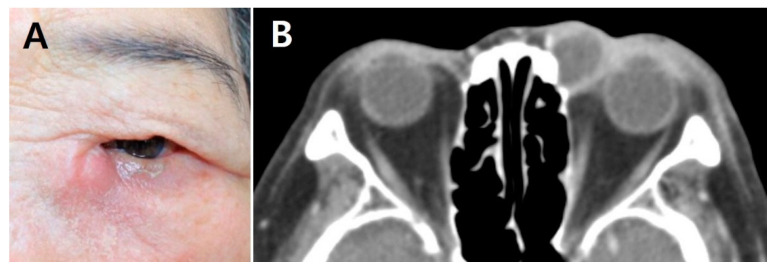
Representative facial photograph and computed tomography (CT) imaging of an AD patient. (**A**) External photograph of an 80-year-old woman who presented with pain, redness, and swelling around left the lacrimal sac area. (**B**) CT scan showing peripheral enhancing cystic lesion in the left medial canthal area with enhancing soft tissue swelling in the adjacent periorbital area.

A retrospective review of medical records was performed on consecutive patients who underwent EnDCR for AD with cellulitis between May 2010 and June 2020 at Chungnam National University Hospital. The diagnosis of AD was based on the clinical presentations with radiologic findings. Patients with painful swelling of the lacrimal sac area with marked medial canthal inflammation were identified. Facial computed tomography was performed in all patients with clinical evidence of AD. Figure 1 shows the representative facial photograph and the computed tomography imaging of an 80-year-old woman with AD. Patients who underwent early EnDCR within 1 week after diagnosis were included. The minimum required follow-up period after surgery was 6 months. Patients who could not be hospitalized on the day of diagnosis were excluded. Patients with a history of lacrimal, maxillofacial, or sinus surgery; facial trauma; or a neoplasm involving the lacrimal drainage system were excluded.

All patients received systemic antibiotics from the time of diagnosis, which continued until the clinical findings of inflammation resolved. The choice of initial empirical intravenous antibiotics were ceftriaxone 1 g every 12 h, amoxicilline/clavulanate 1 g/0.2 g every 6 h, and metronidazole 500 mg every 8 h. Antibiotics were changed according to culture results. The matched control group consisted of patients who underwent EnDCR for primary acquired NLDO by the same surgeon over the same period (non-AD group). Age and gender were matched, and two control patients were randomly selected for each AD patient. Patients with punctal occlusion, canalicular obstruction, history of chronic AD, or a history of lacrimal surgery were excluded. In the non-AD group, patients received prophylactic antibiotics (cefotiam) preoperatively, which were changed to oral antibiotics after surgery.

The study protocol was approved by the Institutional Review Board of Chungnam National University Hospital (IRB no. 2020-12-088) and adhered to the tenets of the Declaration of Helsinki. The requirement for informed patient consent was waived due to the retrospective nature of the study.

### 2.1. Surgical Technique

All operations were performed under general anesthesia by a single experienced surgeon (S.B.L). EnDCR was performed using the technique previously described in [12]. The nasal mucosa was decongested by packing it with 1:10,000 epinephrine-soaked gauze. The upper punctum was dilated, and a 23-gauge vitrectomy illuminator was inserted through the upper canaliculus into the lacrimal sac. After confirming the location of the lacrimal sac in the nasal cavity, 1:100,000 epinephrine plus 2% lidocaine mixed solution was injected into the nasal mucosa of the trans-illuminated area. If necessary, a middle turbinectomy and/or uncinectomy were performed to enlarge the nasal cavity. The nasal mucosa was removed using an elevator and ethmoid forceps. The thick frontal process of the maxillary bone and the thin lacrimal bone were removed using a Smith–Kerrison rongeur. The illuminator was positioned horizontally, tenting the medial wall of the lacrimal sac to ensure that the sac was fully exposed. The lacrimal sac was opened vertically using a crescent knife and was removed using ethmoid forceps. After confirming the patency of the lacrimal passage by saline irrigation, a bicanalicular silicone tube was inserted and the free ends were tied together.

All patients with or without AD were prescribed eye drops (topical antibiotics and topical steroids) and a nasal spray. The silicone tube was removed at 3 months after surgery. The follow-up examinations were scheduled at 1 and 2 weeks, and 1, 2, 3, 4, and 6 months after surgery. At each visit, lacrimal irrigation and ostium evaluation were performed, and patients were asked to report any discomfort.

### 2.2. Main Outcome Measures

The primary outcome measures were the surgical outcomes, including the anatomical and functional success rate at the final follow-up examination. Anatomical success was defined as a patent ostium and was confirmed by lacrimal irrigation without regurgitation. Functional success was defined as the complete resolution of inflammatory symptoms and epiphora. The secondary outcome measure was the clinical course of AD patients, including time for complete resolution of pain and swelling, the length of hospital stay, and the duration of antibiotic treatment. A subgroup analysis was performed according to the timing of surgery. Very early surgery was defined as surgical intervention within 3 days after diagnosis, and early surgery was defined as surgical intervention between 4 and 7 days after diagnosis.

### 2.3. Statistical Analysis

Statistical analysis was performed using SPSS for Windows version 22.0 (SPSS Inc, Chicago, IL, USA). A Student’s *t*-test, a Chi-square test, a Mann–Whitney test, and a Fisher’s exact test were used to compare the patients’ characteristics and surgical outcomes. *p*-values < 0.05 were considered statistically significant.

## 3. Results

### 3.1. Demographics

A total of 41 patients were enrolled in the study group (AD group). The mean age at presentation was 66.9 years (ranging from 37 to 84), and the female to male ratio was approximately 20:1 in the study group. A total of 82 age- and gender-matched patients were identified for the control group (non-AD group). Between the two groups, there were no significant differences in age, gender, laterality, number of patients with a medical history of hypertension and diabetes mellitus, and the postoperative follow-up period. Patient demographics are demonstrated in Table 1.

### 3.2. Surgical Outcomes

At the final follow-up examination, the anatomical and functional success rates were 87.8% and 82.9%, respectively, in the AD group, and 91.5% and 84.1% in the non-AD group. The anatomical and functional success rates were not significantly different between the two groups (*p* = 0.532 and *p* = 0.863) (Table 2). In the AD group, uncinectomy was combined with EnDCR in eight (19.5%) patients, and middle turbinectomy was combined with EnDCR in five (12.5%). There were no significant differences between the two groups in the proportions requiring uncinectomy and/or middle turbinectomy with EnDCR (*p* = 0.245 and *p* = 0.267)

Of the five patients with anatomical failure in the AD group, membranous obstruction of the ostium was observed in three, granulation around ostium in one, and intranasal synechiae in one. No significant complications were noted in the AD group, including severe intraoperative bleeding, delayed severe epistaxis, or recurrent AD.

### 3.3. Clinical Course and Management Outcomes in AD Group

The mean duration of acute symptoms before diagnosis was 7.9 days, and the mean time from diagnosis to operation was 3.8 days. The mean times for pain relief and resolution of swelling after diagnosis were 5.0 days (ranging from 2 to 11 days) and 9.2 days (ranging from 5 to 16 days). The mean times for pain relief and resolution of swelling after surgery were 2.4 days (ranging from 2 to 5 days) and 6.5 days (ranging from 3 to 14 days). The mean durations of hospitalization and antibiotic treatment were 5.5 and 11.0 days. Two patients developed a cutaneous fistula following spontaneous rupture of a lacrimal abscess during antibiotics treatment before surgery. During the follow-up period, all fistulas were closed and no patient reported any concerns about the scar.

### 3.4. Subgroup Analysis According to the Timing of Surgery

The mean duration of acute symptoms before diagnosis was 7.2 ± 4.6 days in the very early EnDCR group and 8.9 ± 5.4 days in the early EnDCR group, which was not significantly different between the two groups. In the very early EnDCR group, pain and swelling resolved within an average of 4.1 and 8.0 days after antibiotic treatment and within 2.4 and 6.3 days after surgery. In the early EnDCR group, pain and swelling resolved on average within 6.5 and 10.9 days after antibiotic treatment and within 2.4 and 6.9 days after surgery. The average length of hospital stay and the duration of antibiotic use were 4.6 days and 9.8 days, respectively, in the very early EnDCR group and 7.0 days and 12.8 days in the early EnDCR group. The time required for symptom resolution after diagnosis, the length of hospital stay, and the duration of antibiotic treatment were significantly shorter after very early EnDCR compared to early EnDCR (all ps < 0.05), whereas the surgical outcomes were not different between the 2 groups (*p* = 1.000) (Table 3).

## 4. Discussion

The conventional treatments of AD consist of warm compresses, systemic antibiotics, and percutaneous abscess drainage, followed by delayed ExDCR. The disadvantages of this approach include a longer time to resolution, prolonged and recurrent infections, fistula formation, cutaneous scar formation, and the risk of failure of subsequent surgery because of scarring, synechiae, and granulation tissue within the lacrimal sac [2,6,7]. Over the past decade, primary EnDCR for the treatment of AD has been reported as a safe procedure with reasonable outcomes [3,4,5,6,7,8,9,10,11,13,14,15,16]. EnDCR allows access to the lacrimal sac through non-infected tissue planes and provides continuous drainage of the lacrimal abscess into the nasal cavity. This can prevent the spread of infection to surrounding tissues and can lead to earlier resolution of the acute symptoms and infection. In addition, a single-stage procedure can reduce the length of hospital stay and the duration of antibiotic treatment without the requirement for subsequent surgery. However, studies analyzing the efficacy of primary EnDCR in large numbers of AD patients are still lacking, and to the best of our knowledge, no studies have explored the optimal timing of EnDCR in AD patients.

The anatomical success rate after primary EnDCR in AD patients varies from 81.8% to 94.4%, and the functional success rate varies from 72.7% to 94.4% [3,4,5]. Duggal et al. [3] analyzed the success rate of EnDCR in 11 patients; the subjective and objective success rates were 72.7% and 81.8%. Madge et al. [4], in a multicenter, non-comparative retrospective study, reported excellent outcomes of primary mechanical EnDCR in 17 patients with AD. At a median follow-up of 12 months, 94.4% of patients were free of epiphora with a patent ostium. Several studies compared the outcomes of EnDCR to those of conventional treatment in patients with AD. Joshi et al. [9] compared the long-term surgical outcomes of 28 EnDCRs and 29 conventional ExDCRs. During a follow-up period of 5 years, the success rates of EnDCR and conventional ExDCR were similar, which were 82.1% and 89.7%, respectively. Wu et al. [5], in a prospective randomized study, compared the outcomes of 40 cold steel EnDCRs and 32 delayed ExDCRs. Ostium patency was achieved in 90% of patients after EnDCR and in 65.7% after ExDCR. Li et al. [2] compared the outcomes of 16 patients who underwent EnDCR as a primary treatment and those of 16 patients who received EnDCR as a secondary treatment after percutaneous drainage of a lacrimal sac abscess. At postoperative year 1, the anatomical and functional success rates were 87.5% in both groups. In our study, the anatomical and functional success rates were 87.8% and 82.9%, comparable with previous reports. These rates were not significantly lower than those of conventional delayed ExDCR, which range from 65.7% to 89.7% [5,9,11]. In addition, the surgical outcomes of primary early EnDCR in AD patients were comparable to those achieved in NLDO patients without AD in our study. The presence of AD at the time of surgery did not seem to be a risk factor affecting the outcomes of primary EnDCR. Previous studies also found that AD was not a risk factor for DCR failure after ExDCR [17]and EnDCR [18] in NLDO patients.

Patients with AD experience acute pain and swelling in the lacrimal sac area. In this study, all patients reported marked pain relief and reduced swelling at postoperative day 1. The mean times for complete pain relief and resolution of swelling were 5 and 9 days, respectively, after systemic antibiotic treatment, and 2 and 7 days after surgery. Our results are consistent with previous studies, in which symptomatic pain relief was usually achieved very early within 1–3 days, and the complete resolution of symptoms was achieved within 5–10 days [5,6,13,15]. Wu et al. [5] reported a shorter time for resolution after primary EnDCR compared to delayed ExDCR in AD patients. The mean times for pain relief and resolution of medial canthal swelling were 1.0 and 3.4 days in the EnDCR group. On the other hand, the mean times were 3.4 and 8.3 days in the ExDCR group. In a recent randomized clinical trial, the time for symptom resolution was 13.5 days in the primary EnDCR group compared to 31.7 days in the secondary EnDCR group after percutaneous drainage [2]. Considering both the clinical course and the surgical outcomes, our results also support that early EnDCR is an effective primary treatment for AD patients.

There may be a difference in the severity of inflammation depending on the course of AD. Previous studies have limitations in suggesting the optimal timing of surgical intervention in AD because of the relatively small sample sizes and wide time intervals from diagnosis to surgery. Recently, Pakdel et al. [11] compared the outcomes of 22 AD patients who underwent very early EnDCR within 3 days after their first visit and those of 19 patients receiving the standard late ExDCR. The overall success rates were comparable between the two groups, being 81.8% in the very early EnDCR group and 89.5% in the late ExDCR group. The average duration of cellulitis in the very early EnDCR group was half that in the late ExDCR group, i.e., 8 and 16 days, respectively. This study suggests that appropriate antibiotic treatment with very early EnDCR within 3 days can be more effective in the resolution of cellulitis.

To evaluate the effects of the timing of surgery in AD patients, we compared the surgical outcomes and clinical course between patients who underwent very early surgery (within 3 days after diagnosis) and early surgery (4 to 7 days after diagnosis). The anatomical success rates (88.0% vs. 87.5%) and functional success rate (84.0 vs. 81.3%) were not significantly different between the two groups (*p* = 1.000 and *p* = 1.000). However, the mean times for pain relief and resolution of swelling after diagnosis were 4.1 and 8.0 days in the very early EnDCR group, significantly shorter than the 6.5 and 10.9 days in the early EnDCR group (*p* = 0.004 and *p* = 0.002). In addition, the length of hospital stay and the duration of antibiotic treatment were also significantly shorter in the very early EnDCR group, at 4.6 and 9.8 days, respectively, compared to 7.0 and 12.8 days in the early EnDCR group (*p* = 0.026 and *p* < 0.001). Although the timing of surgery was not associated with the surgical outcome, the mean time for resolution of acute symptoms, the length of hospital stay, and the duration of antibiotic treatment were significantly shorter after very early surgery. Reducing the time from diagnosis to surgery seems to be important in terms of reducing the treatment period through faster symptom resolution. In addition, a shorter course of antibiotic treatment can reduce the potential risks of drug resistance and drug-related adverse events. When performing primary EnDCR in AD patients, very early surgery within 3 days may have the advantages of shortening the recovery period and reducing the duration of antibiotic treatment.

A common concern when performing DCR in the acute inflammatory stages in the course of AD is that surgery can be challenging because of severe mucosal swelling in the lacrimal sac and canaliculi [2,5]. Increased intraoperative bleeding has been reported in several studies, but usually not to the extent of interfering with the surgery [3,4,6,8,16]. Madge et al. [4] reported mild to moderate bleeding in 28% of patients. In only one case, the procedure was complicated due to excessive bleeding, resulting in poor mucosal flap formation. Difficulties caused by edematous mucosa when introducing either the lacrimal probe or illuminator into the canaliculi have also been reported [3]. Other complications include postoperative epistaxis [13], punctal laceration [5], and tube prolapse [15]. In our study, no remarkable complications were noted. None of the patients showed recurrence of AD during the follow-up period.

The limitations of this study include its retrospective design and the fact that we did not compare the outcomes of primary early EnDCR to those of delayed ExDCR in AD patients. Recent studies on the efficacy of primary EnDCR and our positive experiences have led us to prefer EnDCR as the first-line treatment of AD; therefore, we lacked patients for whom conventional treatments including delayed ExDCR were scheduled. Although a direct comparison with ExDCR was not possible, we believe that our study showed sufficient results supporting the usefulness of EnDCR in the primary treatment of AD. Another limitation is that selecting the timing of EnDCR was not randomized because of the retrospective nature of the study. Surgeries may have been delayed because of the patient’s poor general condition or the use of antiplatelet/anticoagulant agents. Patients with severe cellulitis may not have been able to undergo very early surgery. These factors may affect the difference in the time for symptom resolution between the very early EnDCR group and the early EnDCR group. Recently, Cohen et al. [19] found that smoking was associated with long-term surgical failure in EnDCR. However, in this study, since the number of smokers was too small for statistical comparison, it was not able to reveal the effects of smoking on the surgical outcomes. Further prospective randomized clinical trials are needed to verify our results. Despite these limitations, the strengths of our study include a relatively large sample size and a uniform surgical procedure performed by a single experienced surgeon. Further studies comparing the surgical outcomes of primary early EnDCR performed by surgeons with different degrees of experience may help to generalize our results. In addition, to the best of our knowledge, this is the first study to analyze the effects of surgical timing. Our findings may help clinicians determine treatment options and optimize the timing of interventions in patients with AD.

In conclusion, early EnDCR within 7 days after diagnosis is an effective and safe procedure for the primary treatment of AD. In addition, very early surgery within 3 days of diagnosis reduces the treatment duration and allows for faster recovery without significant complications. If the patient’s general condition is tolerable, very early EnDCR should be considered as a treatment option in patients with AD.

## Figures and Tables

**Table 1:** Demographics of patients.

	AD Group (*n* = 41)	non-AD Group (*n* = 82)	*p* Value
Age (years, mean ± SD)	66.9 ± 12.9	66.8 ± 12.7	0.980 *
Gender (Male/Female, n)	2/39	4/78	1.000 ^†^
Laterality (Right/Left, n)	26/15	43/39	0.248 ^†^
Hypertension (n, %)	18 (43.9)	37 (45.1)	0.881 ^†^
Diabetes mellitus (n, %)	10 (24.4)	14 (17.1)	0.576 ^†^
Follow-up period (years, mean ± SD)	10.0 ± 9.2	9.5 ± 5.3	0.738 *

AD = acute dacryocystitis; SD = standard deviation. * Student’s *t*-test. ^†^ Chi-square test.

**Table 2:** Surgical outcomes of patients.

	AD group (*n* = 41)	non-AD Group (*n* = 82)	*p* Value
Surgical outcomes (n, %)			
Anatomical success rate	36 (87.8)	75 (91.5)	0.532 *
Functional success rate	34 (82.9)	69 (84.1)	0.86 3*

AD = acute dacryocystitis. * Student’s *t*-test.

**Table 3:** Comparison of clinical courses and surgical outcomes between the very early EnDCR group and the early EnDCR group.

	Very Early EnDCR Group (*n* = 25)	Early EnDCR Group (*n* = 16)	*p* Value
Duration of acute symptoms before diagnosis (days, mean ± SD)	7.2 ± 4.6	8.9 ± 5.4	0.291
Clinical courses (days, mean ± SD)			
Resolution of symptoms after diagnosis			
Pain relief	4.1 ± 1.2	6.5 ± 2.7	0.004 *
Resolution of swelling	8.0 ± 2.6	10.9 ± 3.0	0.002 *
Resolution of symptoms after surgery			
Pain relief	2.4 ± 1.0	2.4 ± 1.3	0.968 *
Resolution of swelling	6.3 ± 2.4	6.9 ± 2.0	0.207 *
Length of hospital stay	4.6 ± 2.2	7.0 ± 3.3	0.026 *
Duration of antibiotic treatment	9.8 ± 1.9	12.8 ± 1.8	<0.001 *
Surgical outcomes (n, %)			
Anatomical success rate	22 (88.0)	14 (87.5)	1.000 ^†^
Functional success rate	21 (84.0)	13 (81.3)	1.000 ^†^

AD = acute dacryocystitis; EnDCR = endoscopic dacryocystorhinostomy; SD = standard deviation; * Mann–Whitney test. ^†^ Fisher’s exact test.

## Data Availability

Data supporting the findings of the current study are available from the corresponding author on reasonable request.

## Additional information

Correspondence and requests for materials should be addressed to S.B.L.
